# Investigation of Thermal Behavior of 3D PET Knits with Different Bioceramic Additives

**DOI:** 10.3390/polym12061319

**Published:** 2020-06-09

**Authors:** Audronė Sankauskaitė, Vitalija Rubežienė, Diana Kubilienė, Aušra Abraitienė, Julija Baltušnikaitė-Guzaitienė, Kristina Dubinskaitė

**Affiliations:** 1 Department of Textile Technologies, Center for Physical Sciences and Technology, 48485 Kaunas, Lithuania; audrone.sankauskaite@ftmc.lt (A.S.); ausra.abraitiene@ftmc.lt (A.A.); 2 Department of Textiles Physical-Chemical Testing, Center for Physical Sciences and Technology, 48485 Kaunas, Lithuania; vitalija.rubeziene@ftmc.lt (V.R.); julija.baltusnikaite@ftmc.lt (J.B.-G.); kristina.dubinskaite@ftmc.lt (K.D.)

**Keywords:** 3D textiles, bioceramics, far-infrared radiation, thermoregulatory properties

## Abstract

The purpose of this study is to investigate the thermoregulatory properties of polyethylene terephthalate (PET) 3D knitted materials with bioceramic additives which are highly absorbing far-infrared (FIR) radiation. Ceramic materials are well-known and useful for thermal insulation applications. In order to compare different types of ceramic additives and coating methods for their incorporation into textile, several types of ceramic compounds with heat-retaining function were selected: germanium (Ge), aluminum (Al) and silicon (Si) additives were applied by impregnation in squeezing padder and titanium (Ti) by the screen printing method. The thermoregulatory properties (thermal resistance, heat-retaining effectiveness and air permeability) of 3D PET knits with bioceramic additives were estimated. In this study scanning electron microscopy (SEM) images were used to analyze the morphology of coated fabrics, X-ray fluorescence spectroscopy (XRF) analysis was applied to evaluate the number of minerals with high heat capacity in each formulation used for treatment. The knits coated with a formulation containing Ti ceramic additives demonstrated the most effective thermal behavior. Furthermore, better heat accumulation effectiveness of Ti ceramics containing knits was confirmed by Fourier transform infrared spectroscopy (FTIR) analysis. It was also determined that 3D knitted fabric with Ti ceramic additives showed the highest emissivity among tested samples and the implication is that this sample radiates its energy more efficiently than others.

## 1. Introduction

It is known that fiber type, yarn properties, fabric structure, finishing treatments and clothing wearing conditions are the main factors affecting the thermophysiological comfort of textile fabrics [1,2,3,4]. So-called far-infrared (FIR) textiles are a new category of functional textiles that have presumptive health and wellbeing functionality [5]. The functional component of the FIR textile is bioceramic additives: oxide-based materials with good light reflection such as alumina, zirconium, magnesium, germanium or iron oxides, carbide-based materials with a high heat capacity like ZrC or SiC, photo-catalyst functional materials such as TiO_2_, etc. These additives can emit infrared rays suitable for absorption by the human body, thereby keeping the body warm and accelerating blood circulation. The bioceramic additives as being highly sensitive to far-infrared radiation may considerably influence the thermal behavior of the fabric [6,7,8]. Authors who studied how ceramics incorporated into textile affected their thermal properties [9,10,11], concluded that ceramics absorbed FIR from the body and contained the heat so that the temperature difference between the body and the garment was reduced and the body thus retained its heat better. So, energy from the human body is transferred to these ceramic particles, which are acting as “perfect absorbers”, maintain their temperature at sufficiently high levels and then emit FIR back to the body. 

These additives can be introduced into textile materials in several finishing methods: (1) inserting into the polymer during fiber spinning, (2) impregnating into the fabric through a soaking or coating process. A first method of processing FIR textiles is durable, as ceramic powders are combined with polymer resin, and then the yarn is spun whereby ceramics are incorporated inside the yarn. Applying microceramic particles on the surface of the fiber (using partial penetration as laminate or penetration through the whole fabric volume in terms of soaking), coated or impregnated textile materials are not resistant to the multiple washing and cleaning procedures due to poor fixing of these additives on the textile surface [9]. Another method to incorporate ceramics additives in FIR textiles for thermoregulatory properties improvement was presented in studies [11,12] where Al_2_O_3_, SiO_2_, ZrO_2_ and TiO_2_ were laminated onto breathable hydrophilic polyurethane (PU) films and then incorporated into PET textile material by lamination as an additional layer. As a result, in terms of comfort properties, authors have suggested adding ceramics onto uncoated fabric.

In the IR radiation bands, only FIR transfers energy purely in the form of heat which can be perceived by the thermoreceptors in human skin as radiant heat. FIR (generally λ = 3–100 μm) is a subdivision of the electromagnetic spectrum that was investigated for biological effects. A further subdivision (3–12 μm) of this waveband was observed in both in vitro and in vivo studies, to stimulate cells and tissue, and is considered a promising treatment modality for certain medical conditions [13].

The absorption wavelength range of most organic compounds is 6–14 μm and the human body emits FIR in the range around 10 μm [10]. This is a reason why for investigation of the thermoregulatory effect, the most important wavelength range is 8–12 μm, since it corresponds to thermal radiation at room (or environment) temperature and human body radiation [14]. 

The purpose of this study was to develop and investigate polyethylene terephthalate 3D knitted materials modified with different bioceramic additives; to study the influence of the chemical composition of the ceramic materials used and methods for their incorporation on the enhancement of thermoregulatory properties. As a result, thermal effects, created by ceramic additives integrated into the fabric, have been studied.

## 2. Materials and Methods 

### 2.1. Materials

In this research work investigation of thermal properties of the nonhomogeneous spacer knitted fabrics of complex internal composition with incorporated bioceramic additives was performed. For this purpose, a 3D structure warp-knitted fabric from polyester (PES) and elastane (EL) yarns was used. The main characteristics of the control fabric (code *C)* are presented in Table 1.

In order to improve the thermal efficiency of the spacer fabrics, different bioceramic additives (Ti, Ge, Si-Al) were used applying two coating methods:

- Flat screen printing with Ti mineral containing paste:
  Sample *C1*—fully covered surface;
  Sample *C2*—partly covered surface (squared pattern of a 1 cm^2^ fine line grid);
- Impregnation of textile fabric on squeezing padder:
  Sample *C3*—with Ge mineral containing emulsion;
  Sample *C4*—with Si-Al minerals containing emulsion.

For samples *C1* and *C2*, control fabric was treated with commercially available Ti mineral Paste SCHOELLER ENG PRINT B (Textilcolor AG, Sevelen, Switzerland) applying a flat screen printing method. The main ceramic ingredients in the paste were TiO_2_/MnO/Fe_2_O_3_ which were incorporated into the pigment binder system. The coating was carried out using the laboratory flatbed screen printing device.

**Figure 1:**
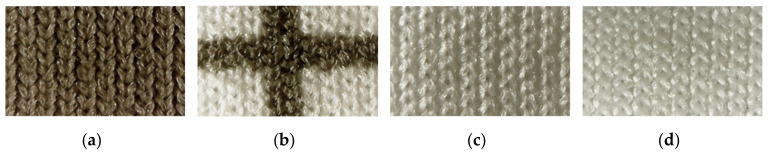
Optical microscope images of treated fabrics outer surface: (**a**) *C1*, (**b**) *C2*, (**c**) *C3*, (**d**) *C4*.

For samples *C3* and *C4*, the control fabric was impregnated with an appropriate mineral mixture containing an aqueous emulsions Itofinish Germanium and Itowarm LJ55 (LJ Specialities Ltd., Chesterfield, Derbyshire, UK), respectively, using a squeezing padder. GeO_2_ was the main ingredient in an aqueous emulsion, used for the *C3* sample. The dominant ingredients in the emulsion, used for *C4*, were SiO_2_/Al_2_O_3_/Na_2_O. Drying–curing was performed in the laboratory oven and steamer machine TFOS IM 350 (Roaches International, Birstall, West Yorkshire, UK). The parameters of impregnation, printing and drying–curing processes are presented in Table 2, and the optical microscope Leica EZ4 (Leica Microsystems, Heerbrugg, Switzerland) images of treated fabrics outer surface are presented in Figure 1.

### 2.2. Methods

#### 2.2.1. XRF Spectroscopy Analysis

X-ray fluorescence spectroscopy (XRF) was applied in order to determine the number of elements with high heat capacity in each composition used in Ti printing paste and Ge, Si-Al aqueous emulsions. Tests were performed applying Bruker X-ray S8 Tiger WD spectrometer (Bruker AXS GmbH, Karlsruhe, Germany): Rh tube was used, anode voltage *U*_a_ under 60 kV, current strength *I* under 130 mA. The measurement was performed using He atmosphere according to the SPECTRA Plus QUANT EXPRESS method. 

#### 2.2.2. SEM Microscopy

The surface morphology of treated fabrics was examined using scanning electron microscopy. The micrographs were taken with SEM Helios Nanolab 650 (FEI, Eindhoven, The Netherlands). A sputter coater was used to pre-coat conductive Pt onto the surface before observing the microstructure at 1.00 kV.

#### 2.2.3. Emissivity Measurements

The IR emissivity of textile fabrics was characterized by means of infrared thermography, a technique which is widely adopted in several fields, and is based on the principle of operation of photothermal techniques [15,16]. Infrared camera, InfraCam (FLIR SYSTEMS, Wilsonville, OR, USA), operating in the midinfrared wavelength range (7.5–13 μm) and providing detailed thermographic images was used. In order to measure IR emissivity following the noncontact procedure, the temperature of the investigated sample, which was placed on an electrically heated plate, was read by the camera (apparent temperature) onto the thermographic image points, and was compared to the temperature read from a reference material of known emissivity deposited onto a portion of the investigated sample. As a reference, black scotch vinyl 33+ electrical tape of emissivity ≈ 0.95 was applied. The IR emissivity of the investigated samples by means of the adopted thermographic method is thus obtained from the ratio of the digital IR signal obtained in correspondence of the sample with respect to that obtained from the reference, whose emissivity value is known. For emissivity calculation software ThermaCAM^TM^ QuickReport, Version 1.0 (FLIR SYSTEMS, Wilsonville, OR, USA) was used.

#### 2.2.4. Infrared Spectroscopy Analysis

To evaluate the absorptivity of investigated fabrics in the FIR range, infrared absorption spectra were measured for textile samples without ceramic additives and textile fabrics with ceramics. For the sample coated with a paste containing Ti minerals (*C1*) there was applied Attenuated Total Reflection (ATR) spectroscopy, as coating with ceramic additives is distributed only on the surface of the fabric. ATR spectroscopy analysis was carried out using the PerkinElmer Frontier IR-FIR spectrometer (PerkinElmer, Inc., Waltham, MA, USA), spectrum range: 600–4000 nm.

For samples impregnated with emulsions of ceramic additives Infrared absorption spectra were measured applying Fourier transform infrared spectroscopy (FTIR) using the KBr Disk technique. For this analysis Infrared spectrophotometer Spectrum BX FT-IR System (PerkinElmer, Inc., Waltham, MA, USA) was used.

#### 2.2.5. Evaluation of Thermoregulatory Properties

Measurements of thermal resistance, air permeability and heat-retaining effectiveness were performed. All these properties were assessed at standard atmosphere: temperature (20 ± 2) °C, relative humidity (65 ± 4)%. 

Thermal resistance was measured under steady-state conditions using sweating guarded-hotplate test according to EN ISO 11092 [17], at temperature (20 ± 2) °C, relative humidity (65 ± 5)%, when the temperature of the hot plate surface was 35 °C. 

Thermal resistance was calculated using Equation (1):

(1) $$R_{ct}=\frac{\left(T_{m}-T_{a}\right)A}{H-\Delta H_{c}}-R_{ct0}$$

where *T_m_* is the temperature of the measuring unit (°C), *T_a_* is the air temperature in the test enclosure (°C), *H* is the heating power supplied to the measuring unit (W), *A* is the area of measuring unit (m^2^), *R_ct_*_0_ is the apparatus constant (m^2^K/W), and Δ*H_c_* is 0 correction term for heating power.

The heat transfer coefficient, *U* (W/m^2^K), was estimated using Equation (2):

(2) $$U=\frac{1}{R_{ct}}$$

Coefficient of thermal conductivity *λ* (W/m K) was calculated using Equation (3):

(3) $$\lambda =\frac{D}{R_{ct}}$$

where *D* is the thickness of sample (m).

Air permeability was measured according to EN ISO 9237 [18], at pressure difference 100 Pa.

**Figure 2:**
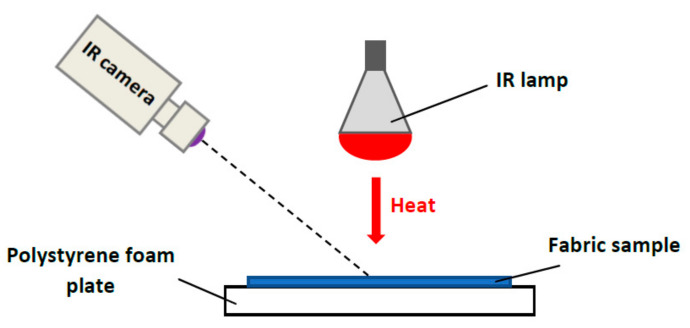
Measurement set-up for evaluation of heat-retaining properties of the fabric.

The heat-retaining properties of PET spacer fabric samples were measured by using a thermal camera (spectral range: λ = 7.5 ÷ 13 μm) InfraCAM and an IR emitting lamp (250 W) as the heat source (Figure 2). At the beginning of the test, the IR lamp was switched on for 4 min to heat up the neat surface of the table. After that period the hottest location was detected by using the InfraCAM. Thereafter, a polystyrene foam plate with a flatly laid spacer fabric sample was centered on the marked hottest location. Samples were subjected to 4 min heating by the IR lamp placed 25 cm over the fabric. Then the lamp was switched off, the fabric was allowed to cool for another 4 min. The samples were observed by the thermal camera every 15 s during the entire 8 min of the test. Five temperature measurements for each sample were performed and the temperature value was calculated. The variation coefficient was <5%.

## 3. Results and Discussion

Tests results of XRF element analysis of commercially available bioceramic additives in printing paste and aqueous emulsions are presented in Table 3. Higher concentrations of elements with high heat capacity were determined in Ge aqueous emulsion (8.60%), where the main ingredient was GeO_2_, and Ti printing paste (5.32%), where the main ingredients were TiO_2_/MnO/Fe_2_O_3_. The dominant ingredients in the Si-Al emulsion were SiO_2_/Al_2_O_3_/Na_2_O, though the quantity of high heat capacity elements in this emulsion was quite low (3.39%).

Based on the results of XRF element analysis, it could be expected that more significant thermal efficiency would be reached in the test fabrics with higher quantity of bioceramic additives, i.e., the higher concentration of high heat capacity elements incorporated into textile leads to higher heat absorbance of the whole fabric. Still, the final results of thermoregulatory properties showed that it is a complex expression of separate factors, which depends not only on quantitative properties but also on the efficiency of different methods of bioceramic additives application (the way where the total amount of the high heat capacity additives is located on the surface of the fabric or it is penetrated through the whole volume). 

**Figure 3:**
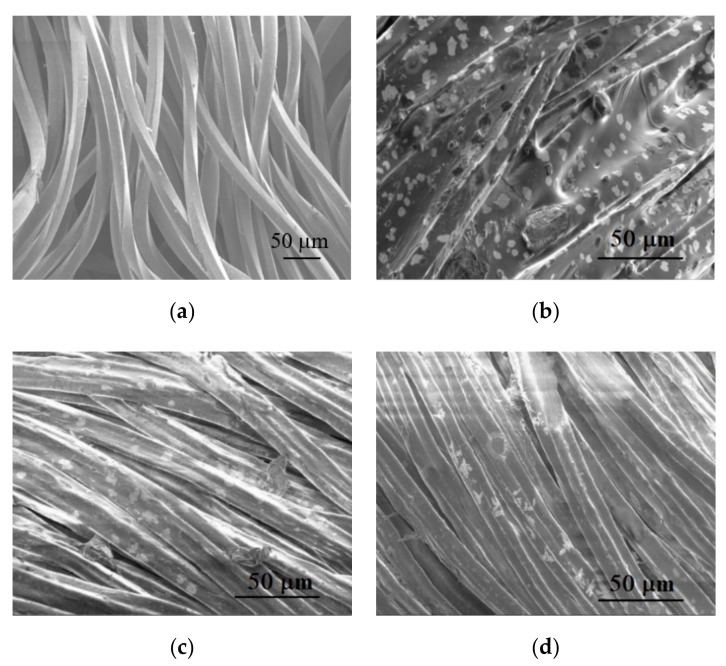
SEM images of the control (**a**) *C* and treated samples: (**b**) *C1*, (**c**) *C3*, (**d**) *C4*.

Therefore, in order to estimate the surface morphology of knits after application of the bioceramic additives the SEM analysis was made. SEM micrographs (Figure 3) show the distribution of ceramic additives on the surface of the fibers and the difference between the surface appearance of fabrics after coating by screen printing (Figure 3b) and after impregnation (Figure 3c,d). It is seen in Figure 3 that the highest amount of bioceramic additives is observed on the knitted surface printed with Ti mineral paste. The printing paste creates a film-like coating (Figure 3b), meaning that ceramic additives are spread only on the surface of the fabric. Comparing SEM images of impregnated fabrics *C3* and *C4* (Figure 3c,d), separate fiber filaments can be observed, that is ceramic additives, in this case, could be distributed throughout the thickness of the fabric. Based on the results of previous studies of researchers [6], it was stated that the entire coating that formed the film on the fabric applying bioceramic additives, led to enhanced thermal properties, but, on the contrary, such thermoregulatory properties as water vapor permeability (breathability) and air permeability were dramatically worse; for example, air permeability was below 0.1 mm/s.

For fabrics developed in our study the lowest air permeability also was obtained in the case of the film formation on the surface (sample *C1*), but it was sufficient, i.e. 414 mm/s, regarding thermo-physiological aspects and enough to declare that fabric was breathable.

The effectiveness of ceramic particles on thermoregulatory properties of fabrics, treated with them, basically is determined by their capability to absorb the FIR rays from the body and to reflect these rays back to the body surface. Regarding these features, ceramic additives incorporated into textiles can modify the optical properties of fabrics. Therefore, in this work, the infrared optical effects, resulting from the incorporation of ceramic additives within knitted 3D structure PET fabric, were evaluated. In particular, changes of emissivity and absorption of fabrics due to the incorporation of different ceramic additives in the FIR range were investigated. 

The measurements of emissivity at 33 ± 2 °C were performed using a thermal camera InfraCAM (spectral range: λ = 7.5 ÷ 13 μm) by means of the adopted thermographic method. Emissivity is a measure of how efficiently an object radiates its energy. It has a scale of values from 1.0 for a perfect emitter, down to 0.0 for materials that do not emit energy [14]. According to Stefan–Boltzmann’s law, the infrared radiation emitted by a hot object is proportional to its emissivity and to the fourth power of its absolute temperature. As a result, the amount of radiation emitted from an object at a given temperature T, is proportional to the emissivity of the surface. Tests results of thermal emissivity measurements (see Table 4) showed that the application of all bioceramic additives used rather increased emissivity, which means also increased the radiation emitted by treated fabrics when they were heated till temperature similar to human body temperature.

The emissivity measurement results presented in Table 4 show that, by including ceramic additives into a 3D knitted fabric, the emissivity can increase over 6%, depending on the composition of ceramic additives and the method of their deposition into the fabric. In particular, for investigated samples obtained an increase in emissivity was: for sample *C1*, 4.4%, for sample *C3*, 6.5% and for sample *C4*, 5.5%. Consequently, among used ceramic additives the best results were obtained in the case of applying Ge aqueous emulsion to sample *C3* (Table 4), though the emissivity values for other treated samples (*C1* and *C4*) were marginally less. For sample *C2* emissivity was not measured as the method used is not suitable for samples with patterns. Comparing with authors [11], who investigated fabrics coated with PU film containing 20% ceramics, we have obtained a slightly lesser increase in emissivity for all tested samples. However, our results are better than those presented by other authors [19] for fabrics made from fibers containing varying amounts of ceramic-bearing polymeric fibers. The results of emissivity measurements are complicated though to match objectively as all mentioned authors used different emissivity measurement methods.

**Figure 4:**
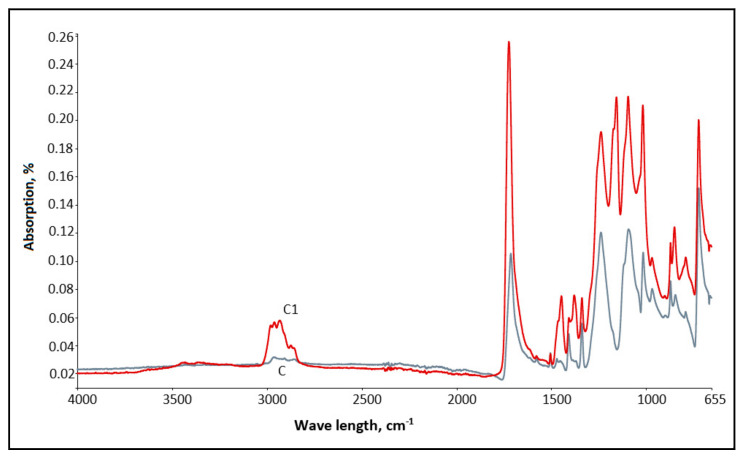
ATR spectrum of samples *C* and *C1*.

To evaluate the capability of investigated fabrics to absorb FIR rays, IR absorption (A,%) spectra, of the control sample (*C*) and textile/bioceramic samples (*C1*, *C3* and *C4*) were measured. The capability of samples to absorb the heat was assessed by IR absorption spectra analysis, focused attention in the subdivision of interest, 8–12 μm (respectively: 1250–833 cm^−1^). The application of ATR and FTIR analysis of investigated samples leads to the identification of the changes of absorption between the control sample and samples with ceramic additives. The obvious and meaningful change in absorptivity, compared with the control sample, was noticed for sample *C1*—fabric coated with Ti mineral containing paste (see Figure 4). Comparing the spectrum of samples *C* and *C1*, the highest difference in absorption was noticed in the range 9.8 ÷ 9.9 μm (respectively: 1020–1010 cm^−1^), which corresponds to the human’s body emitted heat. In this range, the change in absorption between the control sample *C* and sample *C1* seeks more than 0.1%, i.e., absorption values of samples *C* and *C1* at wave range 1010 cm^−1^ were, respectively, 0.094% and 0.175%; and at range 1020 cm^−1^ the values were 0.096% and 0.205% No graphical results are presented for sample *C2* as it is a patterned surface, thereby comparison of absorption might be performed if analyzing separate parts of the sample, i.e., coated (like sample *C1*) and uncoated (sample *C*) areas.

**Figure 5:**
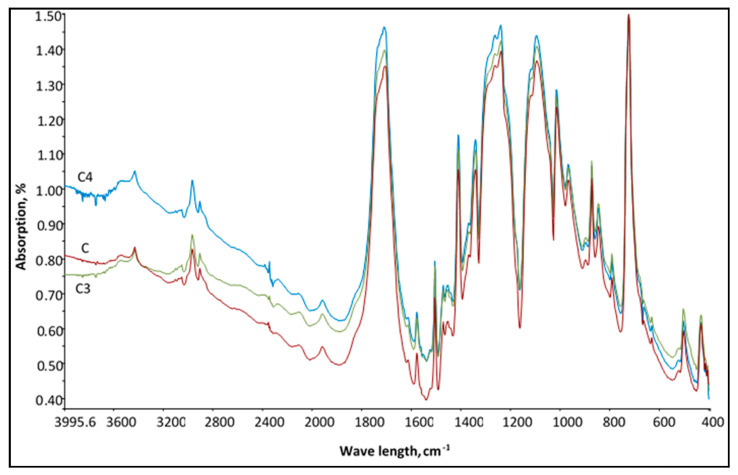
FTIR spectrum of samples *C*, *C3* and *C4*.

For other fabrics with ceramic additives, samples *C3* and *C4*, changes in absorptivity were slightly lesser (see Figure 5). For these samples the highest change in absorption was noticed in the range 11.1 ÷ 11.2 μm (respectively: 900–890 cm^−1^) and was obtained about 0.07%. Absorption values of samples at wave range 900 cm^−1^ were: *C3*—0.871%, *C4*—0.852%, and C—0.801%. Hence, with reference to obtained emissivity and absorption changes, we may confirm that the application of investigated ceramic additives is efficient. The measurements of these optical properties also showed that different ceramic additives used in this study provide the fabric with similar emissivity and absorption in the FIR range. 

**Figure 6:**
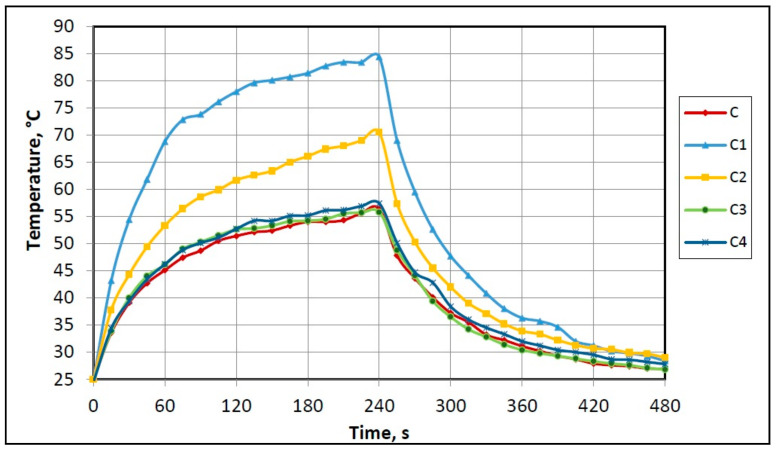
Dynamic thermal behavior of investigated samples.

To evaluate the heat-retaining properties of PET spacer fabric samples, their dynamic thermal behavior when the temperature changes were assessed. These studies (see Figure 6) showed that the best heat storage and slowest heat release was demonstrated by samples with Ti bioceramic paste printed on the whole surface area (*C1*) and squared patterned (*C2*). After irradiance by IR lamp, apparent surface temperature of sample *C1*, fixed by a thermal camera, reached approx. 85 °C; sample *C2*—approx. 72 °C. During the irradiance process, heat accumulation in *C1* significantly increased during the first 60 s and continued increasing till the end of the heating process. Thus, the temperature difference compared to the control sample *C* varied between 24 °C (60 s) and 28 °C (240 s). Analyzing the results of sample *C2*, lesser temperature differences were obtained, respectively, from 8 °C (60 s) to 14 °C (240 s). 

Cooling process analysis showed that the heat retention capability of samples *C1* and *C2* also was better than that of the control sample (*C*) and other samples with ceramics (*C3* and *C4*).

The total dynamic thermal behavior of samples treated with ceramic containing emulsions. Samples *C3* and *C4* practically did not differ from control sample *C* (Figure 6).

Seeing that ceramics can increase thermal insulation by reflecting FIR rays from the body back to the body surface and the body thus retain its heat better [10], evaluation of such thermoregulatory property of fabric as thermal resistance *R*_ct_ may be used to assess the effectiveness of the incorporation of ceramic additives.

The thermal resistance of investigated samples was measured to evaluate their thermal insulating effects. According to measurement results presented in Table 5, it may be stated that in all cases the thermal insulation properties increased. This explains the warming effects of ceramics. 

The results (Table 5) showed that the biggest increase in thermal insulation was obtained for sample *C1*, which surface was fully covered with Ti paste. Other treated fabrics showed only a slight increase in thermal resistance compared to the control sample. Other authors [11] investigated textile fabrics coated with PU film with 20% content of ceramic compounds on the polyester substrate and obtained a similar difference in *R*_ct_ values between the control sample and samples with ceramics, as it is stated for sample *C1* in this study.

Following investigations of thermoregulatory properties, sample *C1*, fully covered with Ti paste, showed the best heat-retaining properties and the highest thermal insulation, though it had the lowermost air permeability. Due to the method of ceramic incorporation for sample *C1*, coating the paste on the surface, air permeability of this sample significantly decreased compared with other samples (Table 5), but was enough to declare that fabric was breathable and could keep the body at a comfortable state. 

Although all investigated samples with ceramic additives showed very similar emissivity and absorption measurement results, their thermal behavior and thermal resistance differed more significantly, especially for sample *C1*. This could be explained by using quite different ceramic incorporation methods, sample *C1* was coated by the screen printing method, where ceramic additives are accumulated on the surface of the fabric, thereby the heat-retaining process is more efficient. Analyzing results of grid coated sample *C2* it could be concluded that partial coating of the fibers (compared to totally coated fibers of sample *C1*) led to lower heat accumulation. 

The influence of the technique of ceramics additives incorporation could be also expressed through test results of samples *C3* and *C4*. Lower thermal accumulation was determined because during impregnation with corresponding emulsions ceramic additives are distributed throughout the thickness of the fabric. Comparing the results of *C2*, *C3* and *C4* in Figure 6, it is evident that higher concentrations of chemical substrate on the fiber surface, only in separate fabric areas (*C2*), showed better heat retention.

## 4. Conclusions

The application of bioceramic particles on the 3D PET fabrics led to the improvement of their thermal efficiency, though resulting in some discussion about the complexity of the total phenomenon.

The comparison of qualitative and quantitative analysis of bioceramic additives showed that thermal efficiency of tested fabrics is a complex expression of separate factors, i.e., a higher concentration of Ge mineral in aqueous emulsion led to a lower quantity of absorbed thermal energy, vs. lower concentration of Ti in the mineral paste, applied as a coating layer (continuous or grid), showed higher results of IR absorption in the subdivision of interest, 8–12 µm (1250–833 cm^−1^). This could be explained by analyzing the efficiency of different methods of bioceramic additives application, which showed that the best heat-retaining properties and the highest thermal insulation were achieved using full surface coating by printing with a paste containing ceramic additives. As ceramic additives are concentrated on the surface of the coated fabric, the heat-retaining process is more efficient compared to the ones impregnated with ceramics containing emulsions, where additives are distributed throughout the thickness of the fabric, thereby leading to lower heat accumulation.

Further study will include an investigation of the resistance performance of fabrics with ceramic additives. This will complement the research works presented in this study as a subsequent, separate publication.

## Figures and Tables

**Table 1:** Characteristics of three-dimensional warp-knitted spacer fabric *C.*

Warp-Wise View of the Fabric	Yarn Type and Linear Density, Tex	Content of Yarn, %
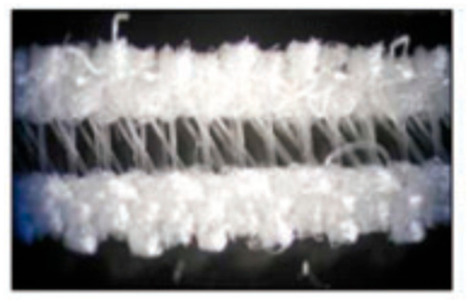	PES textured, 11.0	70
	PES monofilament, 5.6	20
	EL, 7.8	10

**Table 2:** Parameters of impregnation, printing and drying–curing processes.

Process	Auxiliaries	Parameters
Impregnation	Si-Al aqueous emulsion	40 g/L
	Ge aqueous emulsion	50 g/L
	Wet pick-up	80%
	Nip rolls	0.2 MPa
Printing	Ti mineral based paste	Net paste
	Polyester screens mesh	34 threads/cm
	Squeegee angle	70°
	Downforce	1 bar
Drying–curing	Temperature	140 °C
	Time	6 min

**Table 3:** XRF element analysis of main bioceramic additives.

Quantity of Main Elements, wt %					
Ti Printing Paste		Mineral Ge Mixture Aqueous Emulsion		Mineral Si-Al Mixture Aqueous Emulsion	
Ti	2.90	Ge	8.38	Si	2.81
Mn	1.38	Si	0.14	Al	0.43
Fe	1.07	Ca	0.08	Na	0.15
Other elements	3.13	Other elements	0.36	Other elements	1.55
Total	8.48	Total	8.96	Total	4.94
Elements with high heat capacity	5.32	Elements with high heat capacity	8.60	Elements with high heat capacity	3.39

**Table 4:** Emissivity measurement results of investigated samples.

Sample Code	Emissivity (at 33 ± 2 °C)	Standard Deviation
*C*	0.910	0.009
*C1*	0.946	0.009
*C2*	0.946 (coated part)	0.009
	0.910 (no coating)	0.009
*C3*	0.972	0.008
*C4*	0.958	0.008

Note: For each sample, five specimens were measured.

**Table 5:** Measurement results of investigated samples.

Sample Code	Mass per Unit Area *, g/m^2^	Thickness *D* *, mm	Air Permeability *, mm/s	Thermal Resistance *R_ct_* *, m^2^K/W	Heat Transfer Coefficient *U*, W/m^2^K	Thermal Conductivity Coefficient *λ*, W/m K
*C*	372 ± 5	2.7 ± 0.1	618 ± 15	0.063 ± 0.009	15.87	0.043
*C1*	557 ± 8	2.6 ± 0.1	414 ± 30	0.072 ± 0.010	13.89	0.036
*C2*	401 ± 6	2.6 ± 0.1	636 ± 15	0.064 ± 0.009	15.62	0.041
*C3*	375 ± 5	2.7 ± 0.1	641 ± 28	0.065 ± 0.009	15.34	0.042
*C4*	375 ± 5	2.7 ± 0.1	629 ± 17	0.068 ± 0.010	14.71	0.040

Notes: * The reported uncertainty is based on a standard uncertainty multiplied by a coverage factor k = 2, which provides a level of confidence of approximately 95%; *U*, *λ* values are presented without uncertainty as they were estimated from *R_ct_* and *D* measurements.
